# A rise in facial nerve palsies during the coronavirus disease 2019 pandemic

**DOI:** 10.1017/S0022215120002121

**Published:** 2020-10-01

**Authors:** M Zammit, A Markey, C Webb

**Affiliations:** ENT Department, Broadgreen Hospital, Liverpool, UK

**Keywords:** SARS Coronavirus, Facial Palsy, Peripheral Nervous System Diseases

## Abstract

**Objective:**

An increase in spontaneous lower motor neuron facial nerve (VIIth cranial nerve) palsies was seen during the severe acute respiratory syndrome coronavirus 2 outbreak in our emergency clinic. This led us to perform a single-centre cohort review.

**Methods:**

A retrospective review was conducted of VIIth cranial nerve palsies from January to June 2020 and the findings were compared to those cases reviewed in the previous year. The severe acute respiratory syndrome coronavirus 2 incidence of the cohort was compared with that of the Liverpool population.

**Results:**

Our VIIth cranial nerve palsy incidence in the 2020 period was 3.5 per cent (30 out of 852), 2.7 higher than last year's rate of 1.3 per cent (14 out of 1081), which was a statistically significant difference (*p* < 0.01). Two of the 17 patients in our cohort tested positive for severe acute respiratory syndrome coronavirus 2 (11.8 per cent), contrasting with Liverpool's severe acute respiratory syndrome coronavirus 2 incidence (0.5 per cent).

**Conclusion:**

Severe acute respiratory syndrome coronavirus 2 may be responsible for an increased number of facial nerve palsies; it is important for clinicians to be aware that this may being an initial presentation of the disease.

## Introduction

Severe acute respiratory syndrome coronavirus 2 (SARS-CoV-2) is the virus responsible for the current coronavirus disease 2019 (Covid-19) pandemic.^1^ To date, twenty million SARS-CoV-2 cases and over seven hundred thousand deaths have been recorded worldwide.^2^ Emphasis on respiratory symptoms and pulmonary complications has been stressed,^3^ although multi-organ involvement is becoming more apparent from autopsy results.^4^ A retrospective case series of 214 patients, in Wuhan, has confirmed a spectrum of neurological deficits in Covid-19 cases, with both central and peripheral nervous systems involved.^5^

Throughout the Covid-19 outbreak, the authors have maintained daily emergency ENT clinics. We noted a sharp rise in the number of spontaneous lower motor neuron facial nerve (VIIth cranial nerve) palsy cases seen. This has led us to perform a single-centre cohort review on the VIIth cranial nerve palsy patients encountered over the six months from January 2020 to June 2020.

## Materials and methods

Patients with VIIth cranial nerve palsies were retrospectively reviewed from 1st January to 30th June 2020. Patients with a known aetiology for the nerve palsy (such as trauma-induced or Ramsay Hunt syndrome) were excluded from the cohort.

Patients were initially seen in the accident and emergency (A&E) department, Royal Liverpool Hospital, UK, through either self-presentation or a general practitioner referral. Patients identified with an isolated VIIth cranial nerve palsy would be discharged home with medical management and an ENT referral for the emergency clinic.

Patients were contacted for a telephone consultation to reassess their history and symptoms, and to provide a further appointment date in four to six weeks’ time for examination, audiological testing and review of symptom progression. House–Brackmann scoring was utilised to classify severity of the facial palsy on initial examination in A&E and at follow up. Patients were also invited to undergo Covid-19 antibody testing.^6^

The number of VIIth cranial nerve palsies reviewed in the previous year (1st January to 30th June 2019) was established using clinic appointment letters. The latest estimated population size for the city of Liverpool and local number of Covid-19 cases registered up until 30th June 2020 were retrieved from the UK government's online database.^7,8^

The chi-square and McNemar’s tests were used for statistical comparisons, with a probability level of less than 0.05 considered as statistically significant with 1 degree of freedom (95 per cent confidence interval). This analysis was performed using SPSS® statistical software, version 23.

## Results

Thirty-one patients with lower motor neuron VIIth cranial nerve palsy were reviewed in the ENT emergency clinic from 1st January to 30th June 2020. One patient with a diagnosis of Ramsay Hunt syndrome was excluded. All the remaining 30 patients resided in Liverpool during the stipulated time period. The mean age of the cohort was 48 years (range, 18–77 years), with a female-to-male ratio of 1.3:1 and an equal distribution between left- and right-sided facial palsies.

The average duration from symptom onset to presentation at the A&E department was 2.3 days. Four out of the 30 patients (13 per cent) reported a prodromal period of general lethargy, chills and rigors prior to the facial nerve palsy, whilst one patient (3 per cent) reported a preceding episode of nasal congestion. Associated head and neck symptoms are summarised in Table 1. Initial examination in the A&E department confirmed there were no cases of upper motor neuron palsy, with no sparing of the forehead. The average House–Brackmann score was 3.5 (range, 2–5). Computed tomography imaging of the head was performed in four patients (13 per cent) in the emergency setting to rule out a central cause for the nerve palsy; however, no pathologies were detected.

**Table 1.** Associated head and neck symptoms with facial nerve palsy

Associated neurological symptoms	Percentage of cohort (*n*)*
Ipsilateral frontoparietal pain	20 (6)
Ipsilateral post-auricular pain	20 (6)
Dysgeusia	13 (4)
Headaches	10 (3)
Reduced facial sensation	7 (2)
Labyrinthitis & anosmia	3 (1)

* Total *n* = 30

### VIIth cranial nerve palsy management and follow up

Twenty-nine patients (97 per cent) were started on a course of prednisolone. One patient was not prescribed prednisolone, in light of the combination of a mild deficit (House–Brackmann score of 2) and being a type I diabetic. Steroid regimens varied based on clinician preference: 12 patients (40 per cent) received 50 mg daily for 10 days; 12 (40 per cent) had a 10-day tapered regimen commencing with 60 mg daily; 3 patients (10 per cent) were prescribed 60 mg daily for 7 days; and 2 patients (7 per cent) had prednisolone, but there was no documented dosage.

Antiviral therapy was prescribed for eight patients (27 per cent), with four (13 per cent) receiving acyclovir and four (13 per cent) receiving valaciclovir.

Twenty-five patients (83 per cent) were followed up, with five (17 per cent) failing to attend their follow-up clinic appointments. The mean House–Brackmann score on follow up was 1.8 (range, 1–5). Eighteen patients of the 25 patients that attended for follow up (72 per cent) showed a recovery to baseline function (House–Brackmann score of 1). Six patients (24 per cent) had an improvement in House–Brackmann score; however, this was not back to baseline level. Three patients (12 per cent) did not show any improvement and retained their initial House–Brackmann score, with further follow up planned.

### Severe acute respiratory syndrome coronavirus 2 testing

Serological testing for SARS-CoV-2 antibodies was performed in 16 patients (53 per cent), with 1 (3 per cent) yielding a positive Covid-19 antibody test result. Polymerase chain reaction for SARS-CoV-2, via throat swab, was performed in one patient (3 per cent), yielding a positive result.

One of the patients who tested positive had a number of preceding symptoms (labyrinthitis, lethargy, fever, anosmia and post-auricular pain), with a House–Brackmann score of 3 for VIIth cranial nerve palsy, which resolved on a prednisolone tailing regimen. The other patient who tested positive only had a preceding 3-day history of headaches, although the initial House–Brackmann score of 4 for VIIth cranial nerve palsy did not improve on follow up despite a 50 mg prednisolone daily regimen for 10 days.

### Cohort comparisons and statistical findings

**Fig. 1.**
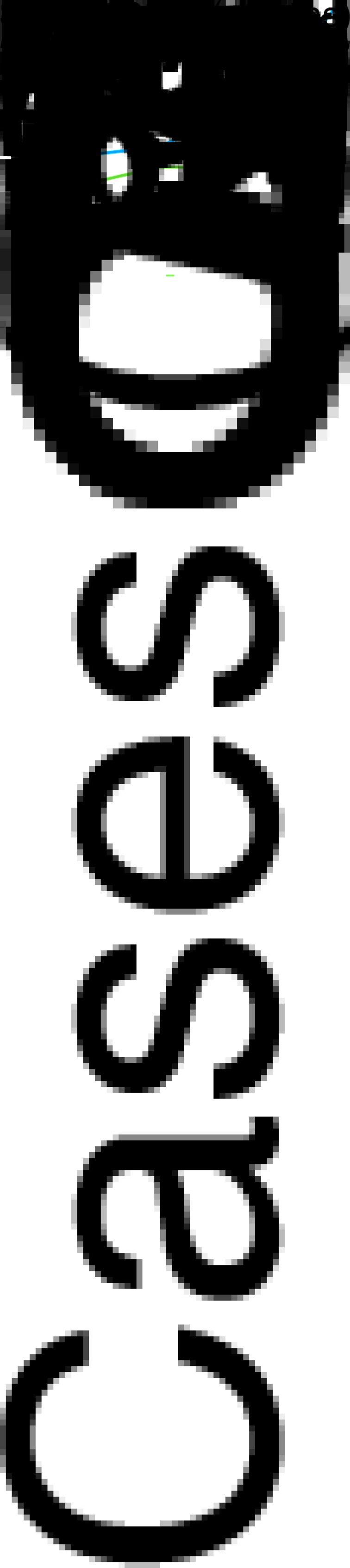
Cumulative cases of facial nerve palsy over six months.

Fourteen patients were diagnosed with VIIth cranial nerve palsy from a total 1081 patients (1.3 per cent) reviewed in the emergency ENT clinic between 1st January 2019 to 30th June 2019. In comparison, 30 patients with VIIth cranial nerve palsy were seen from a total of 852 patients (3.5 per cent) reviewed in the emergency ENT clinic during the same time period in 2020. This represents a 2.7 times increase in incidence. Figure 1 summarises the cumulative number of cases during the respective six-month periods. A chi-square test confirmed a statistically significant difference between the two cohorts, with a *p*-value of 0.002. However, the calculated Cramer's V value, at 0.002, indicated a weak association between the two cohorts.

The latest estimation for the population size of the city of Liverpool is 498 042,^8^ with 2412 recorded Covid-19 cases documented up until 30th June 2020.^7^ This gives an estimated Covid-19 incidence of 0.5 per cent, and contrasts with the incidence of the Covid-19 test subgroup at 11.8 per cent (2 out of 17). McNemar's test revealed a statistically significant increased rate of Covid-19 in our cohort, with a *p*-value of less than 0.001.

## Discussion

Neurological sequelae have been estimated to occur in 36.4 per cent of Covid-19 cases.^5^ Central nervous diseases such as debilitating encephalopathies and ischaemic insults have been described as complications of initial Covid-19 pneumonia.^3,9^ However, a study by Mao *et al*. recognised peripheral nerve involvement, with hypogeusia (82 per cent) and hyposmia (86 per cent) classified as common symptoms of SARS-CoV-2 infection.^5,10^ Ellul *et al*. estimated the number of Covid-19 associated peripheral nervous system diseases to be between 2047 and 7737, based on extrapolations from previous severe acute respiratory syndrome and Middle East respiratory syndrome data.^3^

Furthermore, polyneuropathies including Guillain–Barré syndrome, Miller Fisher syndrome and polyneuritis cranialis have also been reported in infected SARS-CoV-2 patients.^11–14^ Two patients experienced symptoms of Guillain–Barré syndrome as the initial presentation of SARS-CoV-2, and had documented fever 7 days later.^5^

At the time of writing, only two case reports have described a likely correlation between SARS-CoV-2 and isolated cranial neuropathies, specifically VIIth cranial nerve palsy.^15,16^ Both cases reported post-auricular pain as a preceding symptom, and this symptom was recognised in 20 per cent of our cohort. Interestingly, one of the SARS-CoV-2 positive patients experienced a succession of viral-associated symptoms: lethargy and anosmia, followed by labyrinthitis.

The aforementioned polyneuropathies and VIIth cranial nerve palsy are associated with infectious aetiologies.^17^ Whilst studies so far have not shown SARS-CoV-2 nor its family members to be highly neurovirulent,^3^ a number of mechanisms have been postulated.^3,4,10^ Deficits in taste and smell have been seen to occur in isolation of other coryzal symptoms, suggesting possible olfactory bulb involvement and central nervous system invasion.^3,10,16^

However, the virus’ absence in cerebrospinal fluid in a number of Covid-19 cases leads to a different proposition.^3,12,16^ The binding of SARS-CoV-2 to angiotensin-converting enzyme 2 (ACE2) may trigger the body's innate immunological systems to cause elevated pro-inflammatory cytokines and, as a result, neuronal damage.^3,4,10^ ACE2 is also found in the nervous system, therefore implicating a possibly more direct route to nerve injury.^16^

Inflammation-induced demyelination is also a widely supported cause of Bell's palsy, wherein a viral-induced autoimmune reaction results in peripheral demyelination of the cranial nerve. There is a similar high neutrophil-to-leukocyte ratio in Bell's palsy when compared to acute demyelinating diseases (such as Guillain–Barré syndrome); however, the pathways causing this remain unclear.^17^

The significant difference in VIIth cranial nerve palsy cases between the two years in this study suggests a potentially coronavirus-driven increase in palsies.

It is understandably difficult to draw definitive conclusions from differences in the Covid-19 incidences of our cohort and Liverpool's population, given the small patient sample size. However, whilst only 57 per cent of patients (17 out of 30) underwent coronavirus testing, 12 per cent of results (2 out of 17 patients) were positive, which may prove to be an undervaluation if the rest of the cohort underwent coronavirus testing.

Interestingly, Tseng *et al*. showed an association between anxiety disorders and VIIth cranial nerve palsy.^18^ Given the current Covid-19 climate, it may be hypothesised that increased anxiety levels may predispose to VIIth cranial nerve palsies.

With respect to treatment, Wan *et al*. administered prednisolone and the antivirals Arbidol® and ribavirin to their coronavirus-related VIIth cranial nerve palsy patient, who subsequently showed complete recovery.^15^ Conversely, another case of coronavirus-related palsy treated with lopinavir, ritonavir and prednisolone, reported by Goh *et al*., did not display any significant improvement, despite lowering the patient's viral load.^16^ In our cohort, both patients who tested positive for SARS-CoV-2 recovered with solely prednisolone regimens. Antiviral therapy showed indifferent results, with only three of the five patients who were prescribed antivirals and followed up exhibiting a full recovery.

- There was an increased incidence of spontaneous lower motor neuron facial nerve palsy in our emergency ENT clinic
- Only two prior case reports have referenced an association between VIIth cranial nerve palsy and severe acute respiratory syndrome coronavirus 2 (SARS-CoV-2)
- Facial nerve palsy incidence of 3.5 per cent was seen in clinic during 2020, 2.7 times higher than the previous year at 1.3 per cent
- A SARS-CoV-2 incidence of 11.8 per cent was seen in our cohort, contrasting with that of the Liverpool population of 0.5 per cent
- It is important for clinicians to be aware that facial nerve palsy may be an initial presentation of the disease

### Limitations

The major limitation in this study is that the authors were not able to undertake SARS-CoV-2 testing for all the patients in our cohort. Furthermore, six of the serological tests were performed earlier than the ideal six-week waiting period, which may have resulted in an insufficient amount of time for immunoglobulin G SARS-CoV-2 antibodies to be produced, giving a false negative result.

## Conclusion

SARS-CoV-2 has shown its propensity to inflict neurological damage, whether directly or indirectly. Our case series review sheds light on the possibility of Covid-19 causing lower motor neuron VIIth cranial nerve palsy, with or without any preceding Covid-19 symptoms. It is important for clinicians to be aware that this may be an initial presentation of SARS-CoV-2.
